# Blood collection in unstressed, conscious, and freely moving mice through implantation of catheters in the jugular vein: a new simplified protocol

**DOI:** 10.14814/phy2.13904

**Published:** 2018-11-13

**Authors:** Annie Y. Park, Paul M. Plotsky, Truyen D. Pham, Karel Pacak, Brandi M. Wynne, Susan M. Wall, Yoskaly Lazo‐Fernandez

**Affiliations:** ^1^ Department of Medicine Emory University School of Medicine Atlanta Georgia; ^2^ Department of Psychiatry & Behavioral Sciences Emory University School of Medicine Atlanta Georgia; ^3^ Program in Reproductive and Adult Endocrinology Eunice Kennedy Shriver National Institute of Child Health and Human Development National Institutes of Health Bethesda Maryland

**Keywords:** Blood collection, jugular catheter, mice, pendrin, survival surgery

## Abstract

The mouse has become the most common mammalian animal model used in biomedical research. However, laboratory techniques used previously in rats and other larger animals to sample blood had to be adapted in mice due to their lower mouse plasma volume. Sampling is further confounded by the variability in plasma hormone and metabolite concentrations that can occur from the stress or the anesthesia that accompanies the collection. In this article, we describe in detail a protocol we developed for blood sampling in conscious, unrestrained mice. Our protocol implements the use of chronic indwelling catheters in the right external jugular vein, allowing the mice to recover fully in their home cages, untethered until the time of blood sampling. This protocol employs catheters that remain patent for days and does not require the purchase of expensive equipment. We validated this protocol by measuring the time course of plasma norepinephrine (NE) concentration during and after the relief of acute immobilization stress in wild type (WT) and pendrin knockout (KO) mice and compared these results with our previously published values. We found that following relief from immobilization stress, it takes longer for plasma NE concentration to return to basal levels in the pendrin KO than in the wild type mice. These results highlight the potential utility of this protocol and the potential role of pendrin in the neuroendocrine response to acute stress.

## Introduction

Advances in recombinant genetics in the 1990s, and the subsequent development of genetically modified mice, caused a progressive shift from rats to mice as the preferred laboratory animal for biomedical research (Hoyt et al. 2007). This change led to the need to adapt protocols and techniques that had been performed previously in much larger animals. A major challenge has been the miniaturization of protocols employed for repetitive blood sampling. In this article, we describe in detail a protocol we developed and published previously for venous blood sampling in conscious, unrestrained mice. This approach offers a simple, effective, and inexpensive alternative to previous protocols.

Due to the impact of anesthesia and stress, circulating levels of hormones and metabolites can vary significantly depending on the blood collection method employed (Besch et al. 1971; Milakofsky et al. 1984; Conahan et al. 1985; Van Herck et al. 2001; Mattson 2009; Arnold and Langhans 2010; Junuzovic et al. 2011; Flecknell 2016). Affected hormones include stress hormones such as glucocorticoids (Vachon and Moreau 2001; Vahl et al. 2005; Arnold and Langhans 2010; Al‐Noori et al. 2017) and catecholamines (Carruba et al. 1981; Grouzmann et al. 2003; Al‐Noori et al. 2017), although changes in other hormones, electrolytes, and metabolites have also been reported (Carvalho et al. 1975; Philbin and Coggins 1980; Iversen and Andersen 1983; Suzuki et al. 1997; Hauptman et al. 2000; Deckardt et al. 2007). As such, blood collection protocols that can be used in conscious and unstressed animal models are essential for the reliable measurement of blood chemistry parameters, free from the modulating effects of anesthesia or stress.

Blood collection in conscious and unrestrained rats through vessel catheterization has been in wide use since the 1960s (Popovic et al. 1963; Phillips et al. 1973; Migdalof 1976; Burt et al. 1980; Yoburn et al. 1984; Wiersma and Kastelijn 1985; Williams 1985; Gebhardt et al. 2009; Peternel et al. 2010). Catheters are implanted in jugular veins (Picotti et al. 1982; Burvin et al. 1998; Thrivikraman et al. 2002; Tsai et al. 2014) and less frequently in carotid arteries (Yoburn et al. 1984; Heiser and Liu 2007; Feng et al. 2015), femoral arteries or veins (Hall et al. 1984; Williams 1985; Mattson 1998; Jespersen et al. 2012) or tail artery (Fejes‐Tóth et al. 1984; Hagmüller et al. 1992; Balla et al. 2014). Reports of adaptations of these protocols for mice have been comparatively scarce, usually requiring relatively cumbersome surgical steps and the purchase of expensive caging or tethered infusion and/or collection systems (Popovic et al. 1968; Mokhtarian et al. 1993; Mattson 1998; Bardelmeijer et al. 2003; Spoelstra et al. 2004; Mattson 2009), all of which has impaired their widespread use. As a result, we adapted a protocol for the implantation of chronic indwelling catheters in the right external jugular vein in rats (Thrivikraman et al. 2002) for use in mice. This protocol allows the implanted mice to remain free of connecting lines, except for the brief periods of catheter flushing or blood sampling. Moreover, no purchase of specialized or expensive equipment is necessary and the catheters remain patent for many days.

This protocol was used to collect blood samples for catecholamine concentration in mice under basal, unstressed, and stressed conditions to study the role of the Cl^−^/HCO_3_^−^ exchanger pendrin in the function of the adrenal medulla (Lazo‐Fernandez et al. 2015). Since its inception, the protocol has been continuously improved. In this paper, we describe our most updated protocol and demonstrate its effectiveness by comparing the responses of pendrin knockout mice (KO) to wild type littermates (WT) after acute immobilization stress. We anticipate that the relative simplicity and reliability of this protocol will encourage other researchers to learn and use this method of blood sampling in mice.

## Materials and Methods

### Animals

We used 7‐ to 11‐week‐old pendrin (*Slc26a4*) KO (Everett et al. 2001) and their age‐ and gender‐matched WT littermates that were on either a 129 S6SvEvTac or C57Bl/6 backgrounds, Mice were maintained in the vivarium of Emory University under a 12 h light/12 h darkness cycle, in environmentally enriched cages and with ad libitum access to water and food. Starting 3 days prior to surgery, mice were given a balanced diet, prepared as a 27% solid food (Zeigler 538813), 72% water, 0.75% agar (BD, 214010) gel, that was supplemented with NaCl to give each mouse 0.8 meq/day NaCl (Verlander et al. 2003). After surgeries mice were housed in individual cages until blood collection. All procedures are approved by Emory University Institutional Animal Care and Use Committee (IACUC) and comply with NIH (http://grants.nih.gov/grants/olaw/olaw.htm) recommendations based on National Research Council guidelines (National Research Council, 2011).

### Catheters

**Figure 1:**
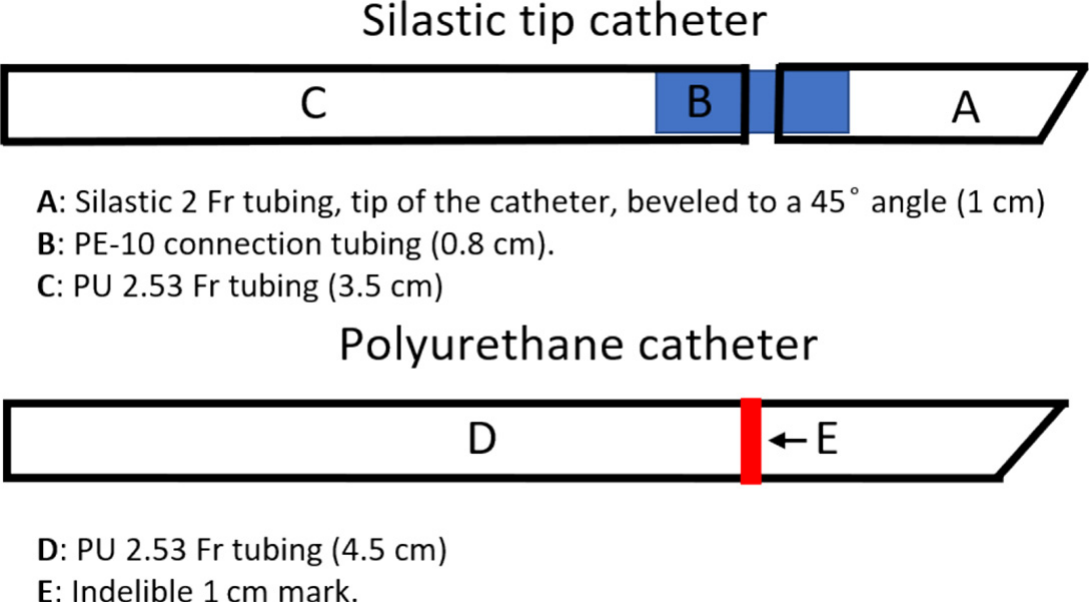
Schematic diagrams of the catheters fabricated and used for experiments.

We first tested the following three different custom‐made or commercial catheters. The first were commercially available polyurethane (PU) mouse catheters obtained from Strategic Applications Inc., USA (SAI, MJC‐01B, MJC‐01B) with a tip outer diameter (OD) of 1 Fr (0.33 mm). The second was a silicone‐tipped catheter (Thrivikraman et al. 2002; Nyuyki et al. 2012), shown in Figure 1, consisting of a 1.1‐cm piece of 2 Fr Silastic tubing (Dow Corning Silastic, Fisher scientific, 11‐189‐14), 0.5‐cm polyethylene (PE) 10 tubing (Intramedic, 427400), and a piece of 2.53 Fr PU tubing (SAI PU‐033). Silastic and PU tubing were immersed in toluene until softened (10–15 sec). PE‐10 was inserted and advanced inside the Silastic or PU tubing. Tubing segments were then cut to the desired lengths and the Silastic tip of the catheter was beveled to a 45° angle to ease the insertion. The third was a simple catheter, (Fig. 1) made entirely from one 4.5 cm long piece of PU 2.53 Fr tubing with the tip beveled to a 45° angle. An indelible mark was made 1 cm from the beveled tip to indicate the approximate length to be inserted in the vein. All catheter tips were smoothed by briefly holding them over the flame from an alcohol burner and then flushed and cleaned with 70% alcohol and sterilized by ethylene oxide gas before use.

### Sterilization of materials

Prior to surgery, all instruments, bench covers, pads, surgical drapes, catheters, and other items were autoclaved or gas sterilized at the Emory University sterilization facility. All packages and pouches for sterilization included a sterilization indicator (3M, Chemical Integrator 1244A). A hot bead sterilizer was used to resterilize instruments during surgeries.

### Surgical and other materials

We employed the following surgical materials and instruments: 3‐0 black braided silk suture (Roboz SUT‐15‐3), Trochar (7 cm long 18G stainless steel beveled tubing), two micro dissecting forceps with 0.8‐mm wide serrated tips (Roboz, RS‐5135); Hartman Mosquito type curved hemostat (Roboz, RS‐7101); vessel dilator forceps (Roboz, RS‐4929); spring‐scissors (Roboz, RS‐5640) and curved iris scissors (Roboz, RS‐5913).

We used polished, blunt 26G 5/8 needles (BD, 26G5/8) to flush solutions through catheters and extension lines. Stainless steel hypodermic tubing adaptors were used to connect catheter and extension lines. Extension lines for blood withdrawal (27–30 cm) and for flushing solutions (9–10 cm) were made of PU 2.53 Fr tubing, since it is flexible and easily fits 26G adaptors, and since its thick wall resists mouse biting or chewing. We also prepared 25‐cm lines of the Silastic 2 Fr tubing for connection to the catheter during surgical insertion to check for the proper position of the catheter. The exteriorized catheter was closed by bending the protruding tubing 180° followed by slipping a 1‐cm piece of PE‐205 or Silastic tubing (1.47 mm ID, Fisher, 11‐189‐15E) over the crimp (Thrivikraman et al. 2002).

A thin, plastic pad (about 23 × 38 cm) was used as a sterile surgical platform placed over the heating pad and a surgical drape. Absorbent bench covers were cut to 12 × 12 cm to be placed on top of the plastic pad. A 30 × 30 cm piece of Press'n Seal food wrap (SAI, 2018) was used as a surgical drape. A rectangular hole at the center provided access to the surgical field. Other items used included: Puralube vet ointment (Dechra, 17033‐211‐38), Vetmond tissue adhesive (3M, 1469SB), Lidocaine Hydrochloride 2% Jelly (Akorn, 17478‐711‐30), as well as alcohol swabs, cotton‐tipped applicators, gauze, and razor blades (all from a variety of vendors).

### Solutions

Cefazolin stock solution was prepared by adding 10 mL of saline to 1 g Cefazolin vials (WG Critical Care, LLC) leading to a 82.5 mg/mL solution. A catheter lock solution containing Cefazolin (4 mg/mL) with heparin (30 IU/mL) in sterile 0.9% saline was prepared before surgery.

### Preparation of the mice

A preoperative dose of Cefazolin 20 mg/kg was administered to each mouse within 2 h of the start of the surgery. An isoflurane saturated induction chamber was used to induce anesthesia. During surgery, mice were anesthetized with 1–2.5% isoflurane/100% O2 gas. Each mouse was transferred to a preparation area where hair was removed, under anesthesia, using a depilatory (NairCare) from the area around the right clavicle and nape of the neck. Each mouse was then placed in a prone position and the area of the back where the catheter would be exteriorized was shaved using an electric hair clipper, then this area was wiped with 70% ethanol and Betadine. Each mouse was then placed in the supine position on the sterile surgical pad, with the head of the mouse pointed towards the surgeon. The forepaws were extended and gently immobilized with tape. The eyes of the mice were protected with sterile ophthalmic ointment. Surgical procedures were performed using sterile technique (Hoogstraten‐Miller and Brown 2008; Pritchett‐Corning et al. 2011). Each animal was placed on a heating pad regulated by a feedback circuit. Catheters were repeatedly flushed with sterile saline prior to use.

### Right external jugular vein isolation

**Figure 2:**
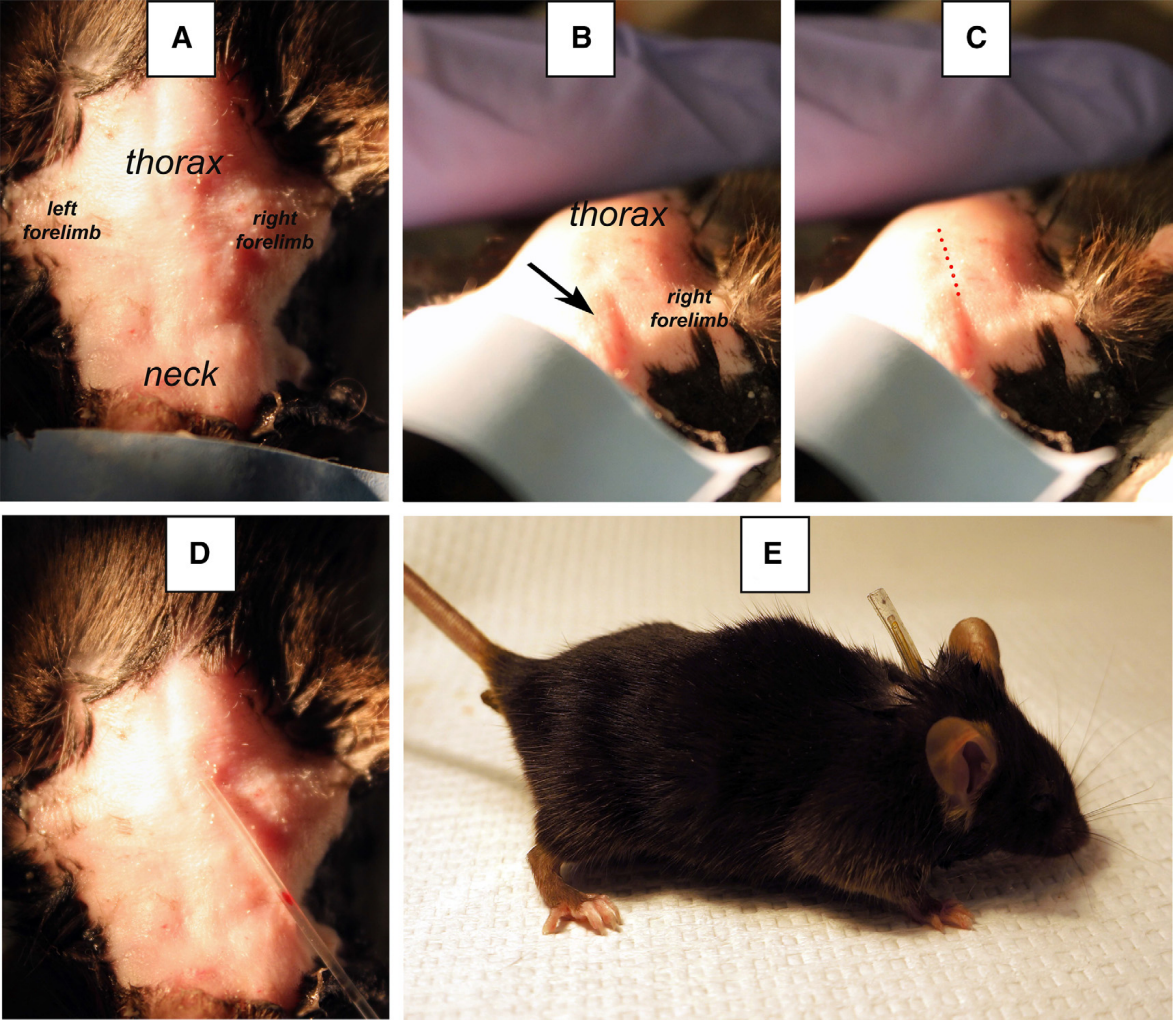
Landmarks used to predict the incision point on the skin above the right external jugular vein of a C57Bl/6 mouse. Panel A shows the surgical area prior to catheter placement with anatomical landmarks indicated. Panel B shows the skin infolding (arrow) used to identify the location of the jugular vein. Panel C shows the approximate path of the jugular vein, indicated by a dotted line. However, the neck shape and the appearance of skin infoldings vary with mouse background strain. Panel D shows how the optimal incision point is located by the indelible mark in the catheter that is placed above the infolding. Panel E shows a catheterized mouse with the capped catheter protruding from its back.

Detailed anatomical characteristics of the jugular veins in rodents are described elsewhere (Cook 1965; MacLeod and Shapiro 1988; Heiser and Liu 2007). The location of the right external jugular vein can sometimes be visualized under bright illumination. However, if the vein was not visible below the shaved skin, we extended an imaginary line along the region where the skin infolds adjacent to the neck (Fig. 2B–D), since the right external jugular vein closely follows this line. An incision was made along this line at about 1 cm from the sternum (Fig. 2). At this point, the skin was elevated using curved forceps and a longitudinal skin incision (6–8 mm length) was made. A 0.4–0.5 cm region of the vein was exposed with adherent tissue removed by blunt dissection with curved forceps, with care to avoid damaging any nerves attached to the vein. This area was kept moist by irrigating the area with sterile saline. If the vein began to collapse or constrict, Lidocaine jelly was applied via sterile cotton‐tipped applicators. The vein was then gently lifted, and a folded 8‐cm sterile suture was slid underneath. The suture was then cut at the bend and the resulting two ligatures were loosely tied around the cranial and caudal portions of the vein. The vein was gently stretched by pulling from these ligatures. The cranial ligature was tightened to interrupt blood flow.

### Catheter implantation

**Figure 3:**
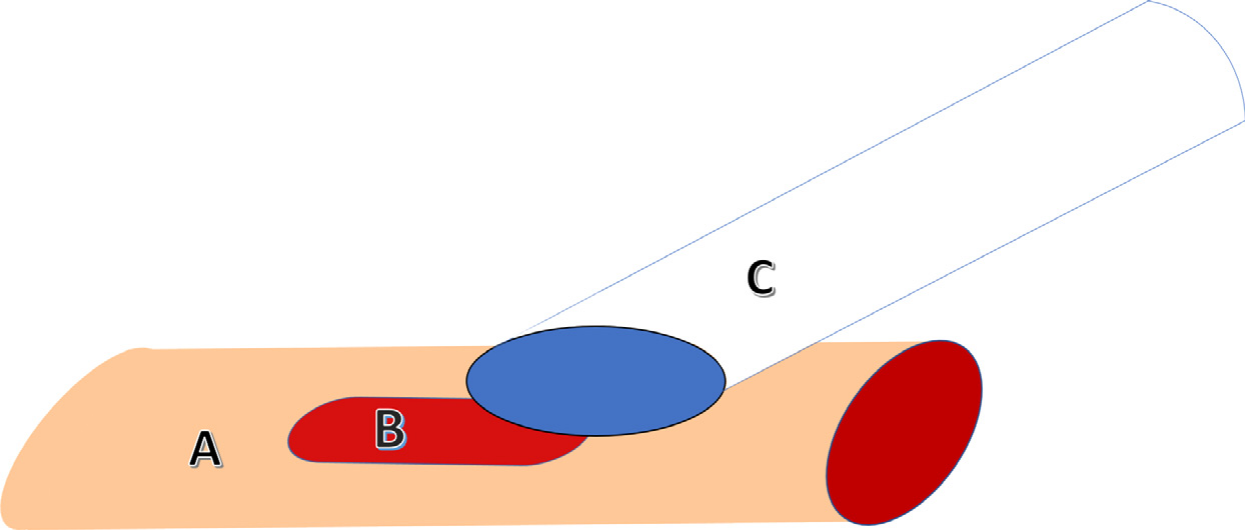
Recommended position of the beveled catheter tip for insertion into the jugular vein. A, jugular vein; B, incision, C, catheter. To cannulate the jugular vein, the beveled side of the catheter tip should approach the incision in the vein sideways.

The rest of the procedure was performed using a surgical microscope with trans‐illumination (Aus Jena Model 212 OPM). The recommended position for the insertion of catheter in the vein is illustrated in Figure 3. A small v‐like incision was made on the wall of the vein, between the ligatures, using microscissors. With the occasional aid of vessel dilator forceps, the beveled end of the catheter was inserted into the jugular vein while gently pulling from the anterior suture to aid in the insertion. After insertion, the catheter was advanced 1 cm into the vein until the tip approximately reached the right atrium. The optimal catheter tip length was determined empirically after performing many surgeries with predominantly young adult mice of both sexes (18–22 g). However, a previous report (Barr et al. 1979) showed that this distance varies in mice from three different strains. As such, the distance between the insertion point on the jugular vein and the atrium changes depending on body size and mouse strain. In our experiments, correct placement was verified by the ease with which blood could be aspirated. If blood could not be withdrawn easily, the catheter was repositioned by advancing or withdrawing it by ~1–2 mm. Once catheter placement was optimized, the caudal suture was tied around the cannulated vein, making sure that blood flow through the catheter remained unrestricted. The catheter was secured to the vein by tightening the cranial ligature a second time, now around the catheter, then flushed with ~20 *μ*L of warm sterile saline.

### Catheter exteriorization

To exteriorize the catheter, each mouse was repositioned on its left side. A sharpened stainless‐steel trochar was tunneled subcutaneously from the ventral incision to the nape of the neck. The catheter was detached from the line, fitted through the trochar, and then exteriorized at the other end. The trochar was then withdrawn from the back. Each mouse was placed in the supine position and the catheter inspected to ensure it was free from kinks or excessive tension. The ventral incision was closed with Vetbond tissue adhesive and swabbed with Betadine. Each mouse was repositioned to a prone position and the catheter was filled with 20 *μ*L of lock solution. The exteriorized end of the catheter was bent at an 180° angle to form a crimp and a small band of tubing was slipped over the crimp to keep it closed so that about 1.5 cm of tubing was protruding upwardly from the back of the mouse. In our observations, keeping this capped segment of the catheter short and straight was essential to restrict the mice's ability to pull the catheter out using their limbs. Still about 5% of implanted mice were able to pull the catheters out. The posterior area of incision was swabbed with Betadine. Meloxicam (5 mg/kg, Loxicom, Norbrook) was administered for analgesia immediately postoperatively and then daily as needed. Each mouse was placed in a clean, warmed, observation cage, and examined every 15 min for about 45 min.

### Flushing the catheters

To maintain patency, catheters were flushed daily with the lock solution (Goossens 2015). To flush the catheters, we modified a technique reported previously for the single‐handed restraint of mice (Hirota and Shimizu 2012). Each mouse was placed on a comfortable soft surface such as a cotton towel or an absorbent pad. In two synchronized movements we seized the tail of the mouse between ring and little finger and applied gentle pressure on the mouse with the other fingers. Once the mouse was immobilized and unagitated, we used the thumb and index fingers of the same hand to grab the catheter protruding from the back of the mouse. With the aid of hemostat or forceps, we used the other hand, to uncap the catheter, connect it to a short extension line or to a syringe, and then infused 10–20 *μ*L of lock solution to verify catheter patency. The catheter was then disconnected and closed.

### Blood sampling

A 30‐cm line of sterile PU 2.53 Fr tubing was connected to the implanted catheter, passed through the wire mesh and partially opened lid of the cage (Super Mouse Microisolation Cage, Lab Products, Inc.), and then secured with adhesive tape to the side of the cage, leaving sufficient tubing length inside for the mouse to move freely in the cage. Each mouse was allowed to rest inside its cage, undisturbed, for about 1 hour prior to blood sampling. Collected blood was processed for NE measurements, as described previously (Lazo‐Fernandez et al. 2015).

### Acute stress test

After 20 min of immobilization stress (Lazo‐Fernandez et al. 2015), the extension line was reconnected, and a blood sample was collected. Each mouse was then transferred back to its home cage for 30 min, after which a final blood sample was obtained. Each mouse was then euthanized, and plasma NE concentration was determined as previously described (Lazo‐Fernandez et al. 2015).

### Hemolysis determination

Blood was collected through a PU catheter, as previously described, and via cardiocentesis. For cardiocentesis, mice were anesthetized with isoflurane, their abdomen and chest wall were opened, and their diaphragm was cut to expose the left ventricle. Blood was aspirated from the left ventricle with an 18‐gauge needle. The mice were then euthanized. Blood samples collected from both methods were allowed to coagulate for 1–2 h at room temperature and serum was collected by centrifugation at 1000*g* for 8 min. Ultraviolet‐visible absorbance at 385 and 414 nm was measured using a NanoDrop 8000 spectrophotometer. The hemolysis score was calculated as described by Appierto et al. (2014).

## Results

### Polyurethane catheters with smoothed tips show improved catheter patency

**Figure 4:**
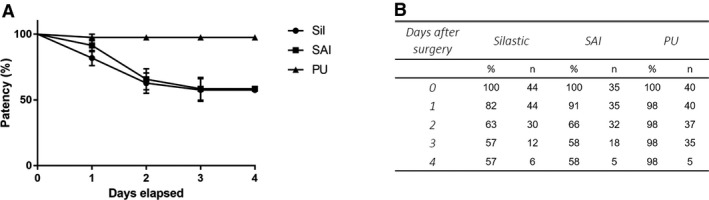
Catheter patency. Panel A shows patency of each catheter tested on postoperative days 1–4. Both SAI (purchased from SAI) and Sil (made by us with Silastic tip) catheters had a 34–37% reduction in patency by the second postoperative day. In contrast, PU catheters showed no reduction in patency over time. The difference in patency rate between the PU catheter curve and the other two catheters was significant (*P* <0.01, log‐rank Mantel‐Cox test). Panel B shows the tabulated data from Panel A, as well as survival proportions (%) and number of mice included in the analysis (n).

Catheter insertion requires a minimum recovery period of 2–3 days before one can collect reliable basal, unstimulated blood samples. In the experiments reported here and in our previous publication (Lazo‐Fernandez et al. 2015), we tested three types of catheters and observed that the patency of the commercial catheters was significantly reduced by the second postoperative day (Fig. 4). We hypothesized that increasing the catheter tip inner diameter (ID) from 0.13 might improve patency. Our initial attempt by constructing catheters with a 2 Fr Silastic tip (0.38 mm ID; Fig. 1) failed to increase patency (Fig. 4).

**Figure 5:**
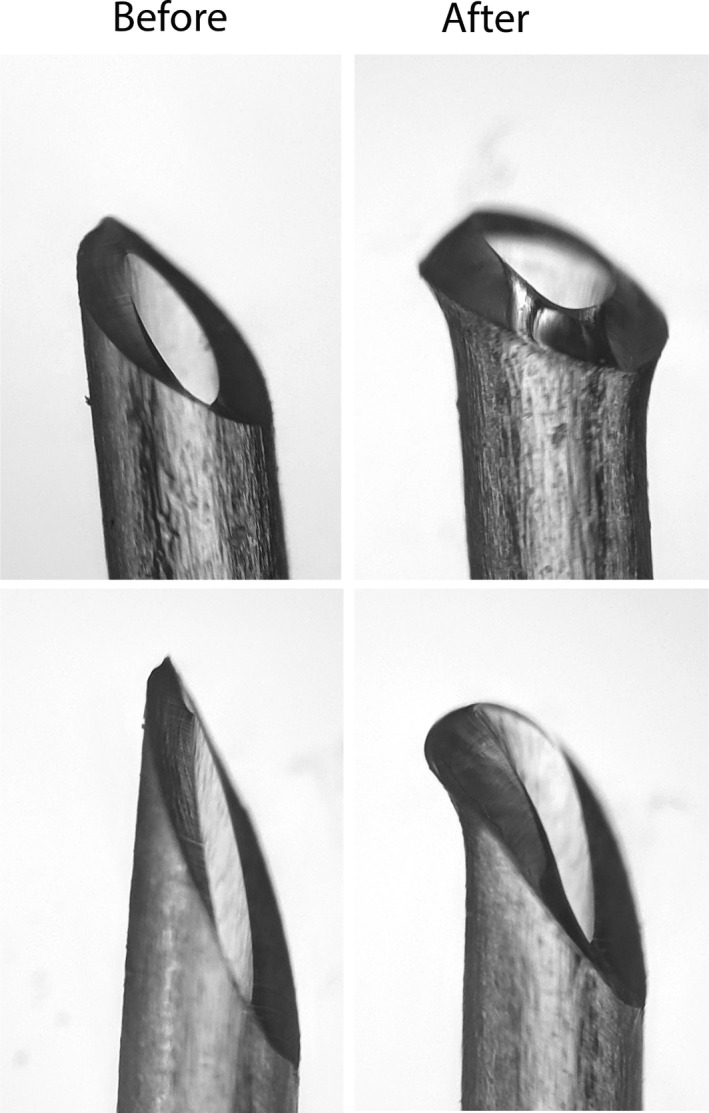
PU catheter tips before and after heat modification.

Since the physical shape of the catheter tip affects thrombogenicity and thus patency (Wickham et al. 1992; Nolan and Klein 2002; Tan et al. 2003), we hypothesized that smoothing the catheter tip would reduce the endothelial injury associated with cannulation. Since Silastic tubing cannot be smoothed by heat, we transitioned to polyurethane (PU) catheters (Fig. 1) and smoothed their tips by flaming the tip with an alcohol burner. Figure 5 illustrates the change in tip shape and smoothness following this procedure. Using the PU catheter, patency 3 days after implantation increased to 98 ± 2% vs. 58 ± 8% with the SAI catheters and 57 ± 8% with the Silastic catheters.

### Hemolysis was not found increased in blood collected using the polyurethane catheters

Hemolysis strongly compromises the accuracy of several medical blood tests (Appierto et al. 2014). The rate of hemolysis in clinical blood samples increases proportionally to the inverse of the cannula inner diameter. As such, significant hemolysis can occur using a 24 gauge cannula or narrower (Kennedy et al. 1996). The tubing used for the PU catheters has an ID of 0.35 mm, which is similar to the ID of a 24‐gauge needle. By visual inspection we did not generally see significant hemolysis in our samples. However many assays are affected by low levels of hemolysis that cannot be detected by the naked eye (Sowemimo‐Coker 2002). Therefore, we quantified hemolysis spectrophotometrically in blood samples collected with our PU catheters as described before. To do so, we compared serum hemoglobin in mouse blood collected either through our jugular catheters or through cardiocentesis using an 18‐gauge needle. We observed no difference in the hemolysis score (Appierto et al. 2014) between samples collected by catheters (0.06 ± 0.01, *n* = 4) versus cardiocentesis (0.07 ± 0.01, *n* = 4). We conclude that the rate of hemolysis is not increased in mouse blood samples collected by our jugular PU catheters, thereby enabling the collection of high quality serum and plasma for biomedical testing.

### NE remains elevated in pendrin null mice 30 min after acute stress

In our previous study (Lazo‐Fernandez et al. 2015), we observed similar catecholamine levels in pendrin null and wild‐type mice under basal, unstimulated conditions, which rose significantly in both groups with immobilization stress. However, after 20 min of immobilization stress, catecholamine concentrations were higher in the mutant (KO) than in wild‐type (WT) mice because catecholamines, and in particular NE, regulate acute changes in blood pressure, we explored the effect of pendrin gene ablation on NE concentration further. More specifically, we asked if plasma NE is higher in pendrin KO relative to WT mice 30 min after termination of immobilization stress. To answer this question, and to validate of our blood collection protocol, we measured plasma NE after 20 min of immobilization stress and then 30 min after termination of the stress, in a group of mice implanted only with PU catheters, and not reported previously (Lazo‐Fernandez et al. 2015).

**Figure 6:**
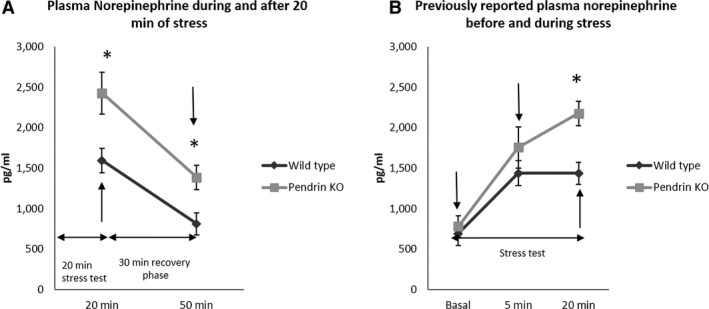
NE measurements in WT and pendrin KO mice. Panel A shows NE levels in mice after 20 min of immobilization stress and then after 30 min of relief from stress during which time they were returned to their cages and were not disturbed. Sampling points are noted with arrows. Plasma NE levels were higher in pendrin KO mice than in WT mice after 20 min of immobilization stress and remained higher 30 min after termination of stress. Wild‐type, *n* = 7; pendrin KO,* n* = 6. Groups were compared with a 2‐tailed, Student's *t*‐test. *P < 0.05. Panel B shows plasma NE levels published previously (Lazo‐Fernandez et al. 2015) and is shown for comparison with the new results in panel A. Blood samples were collected under basal, unstressed conditions and then after 5 and 20 min of immobilization stress (Lazo‐Fernandez et al. 2015).

As shown (Fig. 6) NE was significantly higher (*P* = 0.015) in pendrin KO than in WT mice after 20 min of immobilization stress, similar to our previous observations (Lazo‐Fernandez et al. 2015). In both groups, NE concentration was lower 30 min after cessation of stress, however, NE concentration was still significantly higher in pendrin KO than in the WT mice. This contrasts with our previous observation (Lazo‐Fernandez et al. 2015) that plasma NE concentration is similar in wild type and pendrin null mice under basal, unstressed conditions (Fig. 6). Our results suggest pendrin gene ablation induces a higher plasma concentration of NE in response to acute immobilization stress and a longer poststress recovery period for both blood pressure and plasma NE concentration.

## Discussion

This study represents an extension of our previous work ascertaining the physiological role of pendrin (Wall and Lazo‐Fernandez 2015). In a previous study, we observed that pendrin localizes to the rat, mouse, and human adrenal medulla (Lazo‐Fernandez et al. 2015). To investigate the role of pendrin in adrenal medullary function, we performed sequential blood sampling in conscious WT and pendrin KO mice under basal conditions and then, during and after acute immobilization stress. Toward this objective, we developed reliable and inexpensive implantable jugular catheters for mice that remained patent for at least 2–4 days. While jugular vein catheterization has been used for repeated intravascular administration of substances and blood sampling, maintaining adequate long‐term catheter patency is challenging. Patency maintenance is particularly critical for sampling purposes as the infusion of substances is less restricted by catheter position, fibrin sleeve formation (Hudman and Bodenham 2013), or even vessel occlusion (Figueiredo et al. 2015). Multiple factors influence long‐term patency of intravenous catheters, including catheter material, tip shape, and diameter (Ives 2009; Yang et al. 2016). In the current study, we noted that flame smoothing of the catheter tip increased patency for 2–4 days postinsertion, while increasing catheter diameter was ineffective. While the precise cause of this improvement remains unclear, we speculate that these catheters may cause less endothelial damage, by reducing catheter‐related inflammation (Barraza et al. 2006). We also hypothesize that a more polished surface is less prone to platelet interaction and fibrin coating (Ives 2009). The net result of these two factors would be a reduction in thrombogenicity, in and around the catheter tip, with the resulting reduction of thrombus, fibrin sleeve, and fibrin sheath formation (Forauer et al. 2006). Commercial rodent catheter manufacturers such as SAI (Rohde‐Johnson 2016), Access technologies (Access Technologies, 2018), and Envigo (Envigo, 2018), advertise the advantages of their round tip catheters, largely based on a master's thesis presented in 1995 at Pennsylvania State University (O'Farrell 1995; Nolan and Klein 2002). Our results suggest that designing smoother tips, without sharp edges, represents an effective strategy to reduce catheter thrombogenicity and to increase patency, regardless of the geometry of the tip. While we did not test patency of PU catheters with a slightly smaller OD (e.g. 1.5 to 2 Fr), narrower tips are easier to insert in the vein and, in addition, they tend to cause less vessel injury and allow for greater blood flow around the tip of the catheter (Yang et al. 2016), therefore their use could represent an improvement of this protocol.

Our approach is based primarily on the protocol of Thrivikraman et al. for catheter implantation in rats (Thrivikraman et al. 2002) as well as other reports in mice (Popovic et al. 1968; Barr et al. 1979; MacLeod and Shapiro 1988; Hodge and Shalev 1992; Mokhtarian et al. 1993; Nolan and Klein 2002; Bardelmeijer et al. 2003; Spoelstra et al. 2004; Kmiotek et al. 2012; Nyuyki et al. 2012). Unfortunately, many of those older protocols in mice did not publish sufficient technical detail to enable the techniques to be easily reproduced. While Nyuyki and colleagues published a simplified and more accessible protocol (Nyuyki et al. 2012), their method required dedicated experimental cages and limited blood collection for the first 24 h after catheter implantation. In this report, we describe a detailed protocol for intermediate duration blood sampling over 2–4 days postsurgery. In addition, we describe fabrication of a simple, one‐piece catheter, which provides excellent patency. Our approach also does not require the purchase of specialized experimental caging, vascular access ports, or tethered systems for repeated, manual blood sampling through a chronically indwelling jugular catheter in mice. However, our protocol may not be a viable alternative for all experiments requiring blood sampling in mice. For example, use of vascular access ports is advisable if very frequent samples are to be taken over an extended period of time, when the implanted animals cannot be isolated in their cages, or when using immunosuppressed mice. Tethered systems would be necessary when constant infusions and/or hemodynamic measurements are desired and would be preferred when dual catheterization is intended (Mattson 2009; Jespersen et al. 2012; Feng et al. 2015). Excellent reviews on the use of vascular access ports and tethered systems are available elsewhere (Nolan and Klein 2002; Nolan et al. 2008).

For technical verification of our protocol, we measured plasma NE concentration in blood samples collected 2–4 days after the insertion of a venous catheter in WT and pendrin KO mice following 20 min of acute immobilization stress and then 30 min after relief from stress. Both groups of mice had a high plasma NE concentration during immobilization stress, which then fell with the relief of stress (Fig. 6). These new data confirmed our previous observations that plasma NE was higher in pendrin KO vs WT mice following 20 min of immobilization stress (Lazo‐Fernandez et al. 2015). However, whereas our previous study showed that plasma NE concentration is similar in pendrin null and wild type mice under basal, unstimulated conditions, the present study shows that 30 min after the relief of stress, plasma NE concentrations differ in these mice. In wild type mice, NE levels were similar to those observed under unstimulated conditions while in pendrin null mice, NE concentration remained elevated for longer following relief from stress. As such, 30 min after the relief of stress, plasma NE concentration remained higher in the pendrin null mice than in wild type mice. These results were compared with the blood pressure measured previously in pendrin null and wild type mice, under basal conditions, following 20 min of immobilization stress and then 30 min after relief from stress (Lazo‐Fernandez et al. 2015). We observed that blood pressure rose with immobilization stress in both groups. However, 30 min following relief from stress mean arterial pressure had returned to basal levels in WT mice, but not in KO mice (Lazo‐Fernandez et al. 2015). These data indicate that following the relief of stress, it takes longer for NE concentration and blood pressure to fall to basal levels in the pendrin KO than in wild type mice.

Circulating NE produces vasoconstriction via *α*1 receptors, leading to an acute increase in total peripheral resistance and arterial blood pressure (Barrett et al. 2016). In response to the application of an *α* agonist (phentolamine) in vitro, contractile force of the thoracic aorta is greater in pendrin null than in wild type mice (Sutliff et al. 2014). Consequently, the higher NE levels observed in pendrin KO mice 30 min after relief of stress might explain why blood pressure remains elevated in pendrin KO mice after 30 min of recovery from stress relative to basal, unstimulated levels (Lazo‐Fernandez et al. 2015). Why pendrin deletion results in a slower return of NE to basal levels will be the topic of future studies.

In summary, we report a simplified and inexpensive protocol for blood sampling through catheter implantation in the mouse's right external jugular vein. Our catheters are easy to fabricate and remain patent for at least 3–4 days postsurgery. Overall, this protocol is relatively straightforward and does not entail the use of costly equipment or supplies. It allows for precisely timed, sequential blood sampling in conscious, unrestrained mice under basal (or pretreatment), and stimulated conditions, permitting the determination of the time‐course in response to a treatment. In so doing, it should increase the accuracy of such studies as well as reduce the number of animals needed for these studies.

## Conflict of Interest

The authors have no conflict of interests to disclose.

## References

[phy213904-bib-0001] Access Technologies . 2018 Catheters [Internet]. Available from: http://www.norfolkaccess.com/catheters.html

[phy213904-bib-0002] Al‐Noori, S. , A. Cimpan , Z. Maltzer , K. Kaiyala , and D. Ramsay . 2017 Plasma corticosterone, epinephrine, and norepinephrine levels increase during administration of nitrous oxide in rats. Stress 21:274–278. 29145764 10.1080/10253890.2017.1402175 PMC6310116

[phy213904-bib-0003] Appierto, V. , M. Callari , E. Cavadini , D. Morelli , M. Daidone , and P. Tiberio . 2014 A lipemia‐independent NanoDrop^®^‐based score to identify hemolysis in plasma and serum samples. Bioanalysis 6:1215–1226. 24946922 10.4155/bio.13.344

[phy213904-bib-0004] Arnold, M. , and W. Langhans . 2010 Effects of anesthesia and blood sampling techniques on plasma metabolites and corticosterone in the rat. Physiol. Behav. 99:592–598. 20152845 10.1016/j.physbeh.2010.01.021

[phy213904-bib-0005] Balla, D. , S. Schwarz , H. Wiesner , A. Hennige , and R. Pohmann . 2014 Monitoring the stress‐level of rats with different types of anesthesia: a tail‐artery cannulation protocol. J. Pharmacol. Toxicol. Methods 70:35–39. 24632523 10.1016/j.vascn.2014.03.003

[phy213904-bib-0006] Bardelmeijer, H. , T. Buckle , M. Ouwehand , J. Beijnen , J. Schellens , and O. Tellingen . 2003 Cannulation of the jugular vein in mice: a method for serial withdrawal of blood samples. Lab. Anim. 37:181–187. 12869279 10.1258/002367703766453010

[phy213904-bib-0007] Barr, J. E. , D. B. Holmes , L. J. Ryan , and S. K. Sharpless . 1979 Techniques for the chronic cannulation of the jugular vein in mice. Pharmacol. Biochem. Behav. 11:115–117. 493295 10.1016/0091-3057(79)90307-1

[phy213904-bib-0008] Barraza, M. , J. Strickland , H. Zepeda , J. Taylor , C. Krehbiel , G. Bell , et al. 2006 Gross and histopathological observations of long‐term catheterized vessels in experimental sheep. J. Vet. Med. A Physiol. Pathol. Clin. Med. 53:230–238. 16737512 10.1111/j.1439-0442.2006.00824.x

[phy213904-bib-0009] Barrett, K. E. , S. M. Barman , S. Boitano , and H. L. Brooks . 2016 The adrenal medulla & adrenal cortex in Ganong's review of medical physiology [Internet]. McGraw‐Hill Education, New York, NY Available from: http://accessmedicine.mhmedical.com/content.aspx?aid=1115830237

[phy213904-bib-0010] Besch, E. , B. Chou , and C. Cornelius . 1971 Physiological responses to blood collection methods in rats. Proc. Soc. Exp. Biol. Med. Soc. Exp. Biol. Med. 138:1019–1021. 10.3181/00379727-138-36041 5131601

[phy213904-bib-0011] Burt, M. , J. Arbeit , and M. Brennan . 1980 Chronic arterial and venous access in the unrestrained rat. Am. J. Physiol. 238:H599–H603. 7377334 10.1152/ajpheart.1980.238.4.H599

[phy213904-bib-0012] Burvin, R. , M. Zloczower , and E. Karnieli . 1998 Double‐vein jugular/inferior vena cava clamp technique for long‐term in vivo studies in rats. Physiol. Behav. 63:511–515. 9523892 10.1016/s0031-9384(97)00486-1

[phy213904-bib-0013] Carruba, M. , G. Picotti , P. Miodini , W. Lotz , and M. Prada . 1981 Blood sampling by chronic cannulation technique for reliable measurements of catecholamines and other hormones in plasma of conscious rats. J. Pharmacol. Meth. 5:293–303. 10.1016/0160-5402(81)90041-3 7311568

[phy213904-bib-0014] Carvalho, J. , R. Shapiro , P. Hopper , and L. Page . 1975 Methods for serial study of renin‐angiotensin system in the unanesthetized rat. Am. J. Physiol. 228:369–375. 1119559 10.1152/ajplegacy.1975.228.2.369

[phy213904-bib-0015] Conahan, S. , S. Narayan , and W. Vogel . 1985 Effect of decapitation and stress on some plasma electrolyte levels in rats. Pharmacol. Biochem. Behav. 23:147–149. 4034618 10.1016/0091-3057(85)90143-1

[phy213904-bib-0016] Cook, M. J . 1965 Branches of the subclavian vein. In: The anatomy of the laboratory mouse [Internet]. Adapted for the Web by Mouse Genome Informatics, The Jackson Laboratory, Bar Harbor, Maine; 1965. p. 98. Available from: http://www.informatics.jax.org/cookbook/figures/figure98.shtml

[phy213904-bib-0017] Deckardt, K. , I. Weber , U. Kaspers , J. Hellwig , H. Tennekes , and B. Ravenzwaay . 2007 The effects of inhalation anaesthetics on common clinical pathology parameters in laboratory rats. Food Chem. Toxicol. 45:1709–1718. 17459552 10.1016/j.fct.2007.03.005

[phy213904-bib-0018] Envigo . 2018 Rounded‐tip catheter [Internet]. Available from: http://www.envigo.com/resources/surgical-sheets/envigo-89-rounded-tip-catheter_screen.pdf

[phy213904-bib-0019] Everett, L. , I. Belyantseva , K. Noben‐Trauth , R. Cantos , A. Chen , S. Thakkar , et al. 2001 Targeted disruption of mouse Pds provides insight about the inner‐ear defects encountered in Pendred syndrome. Hum. Mol. Genet. 10:153–161. 11152663 10.1093/hmg/10.2.153

[phy213904-bib-0020] Fejes‐Tóth, G. , A. Náray‐Fejes‐Tóth , D. Ratge , and J. Frölich . 1984 Chronic arterial and venous catheterization of conscious, unrestrained rats. Hypertension 6:926–930. 6394488 10.1161/01.hyp.6.6.926

[phy213904-bib-0021] Feng, J. , Y. Fitz , Y. Li , M. Fernandez , I. Puch , D. Wang , et al. 2015 Catheterization of the carotid artery and jugular vein to perform hemodynamic measures, infusions and blood sampling in a conscious rat model. JoVE 95:51881. 10.3791/51881 PMC4354559 25741606

[phy213904-bib-0022] Figueiredo, G. , T. Fiebig , S. Kirschner , O. Nikoubashman , L. Kabelitz , A. Othman , et al. 2015 Minimally invasive monitoring of chronic central venous catheter patency in mice using digital subtraction angiography (DSA). PLoS ONE 10:e0130661. 26098622 10.1371/journal.pone.0130661 PMC4476576

[phy213904-bib-0023] Flecknell, P . 2016 Basic principles of Anaesthesia Pp.1–75 in Laboratory animal Anaesthesia. Academic Press, Cambridge, MA [cited 2020]. Available from: https://www.sciencedirect.com/science/article/pii/B9780128000366000016

[phy213904-bib-0024] Forauer, A. R. , C. G. Theoharis , and N. L. Dasika . 2006 Jugular vein catheter placement: histologic features and development of catheter‐related (Fibrin) sheaths in a Swine Model. Radiology 240:427–434. 16864670 10.1148/radiol.2402031129

[phy213904-bib-0025] Gebhardt, H. , F. Fandrich , H. Schaube , J. Schröder , and E. Deltz . 2009 A novel technique for long‐term vascular access in the unrestrained rat. J. Invest. Surg. 8:425–431. 10.3109/08941939509031608 8751153

[phy213904-bib-0027] Goossens, G. 2015 Flushing and locking of venous catheters: available evidence and evidence deficit. Nurs. Res. Pract. 2015:1–12. 10.1155/2015/985686 PMC4446496 26075094

[phy213904-bib-0028] Grouzmann, E. , C. Cavadas , D. Grand , M. Moratel , J.‐F. Aubert , H. R. Brunner , et al. 2003 Blood sampling methodology is crucial for precise measurement of plasma catecholamines concentrations in mice. Pflugers Arch. 447:254–258. 12905032 10.1007/s00424-003-1140-x

[phy213904-bib-0029] Hagmüller, K. , P. Liebmann , S. Porta , and I. Rinner . 1992 A tail‐artery cannulation method for the study of blood parameters in freely moving rats. J. Pharmacol. Toxicol. Methods 28:79–83. 1482789 10.1016/1056-8719(92)90051-2

[phy213904-bib-0030] Hall, R. I. , L. H. Ross , M. Bozovic , and J. P. Grant . 1984 A simple method of obtaining repeated venous blood samples from the conscious rat. J. Surg. Res. 36:92–95. 6690846 10.1016/0022-4804(84)90072-6

[phy213904-bib-0031] Hauptman, J. , M. Richter , S. Wood , and R. Nachreiner . 2000 Effects of anesthesia, surgery, and intravenous administration of fluids on plasma antidiuretic hormone concentrations in healthy dogs. Am. J. Vet. Res. 61:1273–1276. 11039560 10.2460/ajvr.2000.61.1273

[phy213904-bib-0032] Heiser, A. , and J. H. K. Liu . 2007 Rat jugular vein and carotid artery catheterization for acute survival studies. Springer‐Verlag, New York.

[phy213904-bib-0033] Hirota, J. , and S. Shimizu . 2012 Routes of administration Pp. 709–725 in Hedrich H. J., ed. The laboratory mouse. 2nd ed Elsevier, Academic Press, Cambridge, MA.

[phy213904-bib-0034] Hodge, D. E. , and M. Shalev . 1992 Dual cannulation: a method for continuous intravenous infusion and repeated blood sampling in unrestrained mice. Lab. Anim. Sci. 42:320–322. 1320170

[phy213904-bib-0035] Hoogstraten‐Miller, S. L. , and P. A. Brown . 2008 Techniques in aseptic rodent surgery. Curr. Prot. Immunol. 82:1.12.1–1.12.14. 10.1002/0471142735.im0112s82 PMC2587003 18729061

[phy213904-bib-0036] Hoyt, R. E. , J. V. Hawkins , M. B. St Clair , and M. J. Kennett . 2007 Mouse physiology Pp. 23–90 in Fox J. G., Davisson M. T., Quimby F. W., Barthold S. W., Newcomer C. E., Smith A. L., ed. The mouse in biomedical research. 2nd ed Academic Press, Cambridge, MA Available from: https://www.sciencedirect.com/science/article/pii/B978012369454650056X

[phy213904-bib-0037] Hudman, L. , and A. Bodenham . 2013 Practical aspects of long‐term venous access. BJA Educ. 13:6–11.

[phy213904-bib-0038] Iversen, B. , and K. Andersen . 1983 The effect of sampling conditions on rat plasma renin. Comp. Biochem. Physiol. Part A: Physiol. 74:331–332. 10.1016/0300-9629(83)90611-4 6131777

[phy213904-bib-0039] Ives, F . 2009 Catheter design and materials Pp. 57–77 in Hamilton H. and Bodenham A. R., ed. Central venous catheters. Wiley‐Blackwell, Hoboken, NJ.

[phy213904-bib-0040] Jespersen, B. , L. Knupp , and C. Northcott . 2012 Femoral arterial and venous catheterization for blood sampling, drug administration and conscious blood pressure and heart rate measurements. J. Vis. Exp. (59):e3496 10.3791/3496. PMC3462562 22297665

[phy213904-bib-0041] Junuzovic, D. , E. Celic‐Spuzic , and M. Hasanbegovic . 2011 The correlation between type of anesthesia and the hormones levels during and after transvesical prostatectomy. Acta Inform. Med. 19:216–219. 23408784 10.5455/aim.2011.19.216-219 PMC3564185

[phy213904-bib-0042] Kennedy, C. , S. Angermuller , R. King , S. Noviello , J. Walker , J. Warden , et al. 1996 A comparison of hemolysis rates using intravenous catheters versus venipuncture tubes for obtaining blood samples. J. Emerg. Nurs. 22:566–569. 9060320 10.1016/s0099-1767(96)80213-3

[phy213904-bib-0043] Kmiotek, E. K. , C. Baimel , and K. J. Gill . 2012 Methods for intravenous self administration in a mouse model. JoVE 70:3739. 10.3791/3739 PMC3567158 23242006

[phy213904-bib-0044] Lazo‐Fernandez, Y. , G. Aguilera , T. D. Pham , A. Y. Park , W. H. Beierwaltes , R. L. Sutliff , et al. 2015 Pendrin localizes to the adrenal medulla and modulates catecholamine release. Am. J. Physiol. Endocrinol. Metab. 309:E534–E545. 26173457 10.1152/ajpendo.00035.2015 PMC4572452

[phy213904-bib-0045] MacLeod, J. , and B. Shapiro . 1988 Repetitive blood sampling in unrestrained and unstressed mice using a chronic indwelling right atrial catheterization apparatus. Lab. Anim. Sci. 38:603–608. 3193754

[phy213904-bib-0046] Mattson, D. 1998 Long‐term measurement of arterial blood pressure in conscious mice. Am. J. Physiol. 274(2 Pt 2):R564–R570. 9486319 10.1152/ajpregu.1998.274.2.R564

[phy213904-bib-0047] Mattson, D. L . 2009 Measuring kidney function in conscious mice Pp. 75–94 in Cardiovascular genomics methods in molecular biology™ (Methods and Protocols) vol 573. Humana Press, Totowa, NJ Available from: https://link.springer.com/protocol/10.1007/978-1-60761-247-6_5#citeas 10.1007/978-1-60761-247-6_5 19763923

[phy213904-bib-0048] Migdalof, B. 1976 Methods for obtaining drug time course data from individual small laboratory animals: serial microblood sampling and assay. Drug Metab. Rev. 5:295–310. 802470 10.3109/03602537609029981

[phy213904-bib-0049] Milakofsky, L. , T. Hare , J. Miller , and W. Vogel . 1984 Comparison of amino acid levels in rat blood obtained by catheterization and decapitation. Life Sci. 34:1333–1340. 6708734 10.1016/0024-3205(84)90004-3

[phy213904-bib-0050] Mokhtarian, A. , M. Meile , and P. Even . 1993 Chronic vascular catheterization in the mouse. Physiol. Behav. 54:895–898. 8248379 10.1016/0031-9384(93)90298-t

[phy213904-bib-0051] National Research Council . 2011 National Research Council (US) Committee for the update of the guide for the care and use of laboratory animals. Guide for the care and use of laboratory animals [Internet]. 8th ed National Academies Press (US), Washington, DC Available from: https://www.ncbi.nlm.nih.gov/books/NBK54050/

[phy213904-bib-0052] Nolan, T. , and H. Klein . 2002 Methods in vascular infusion biotechnology in research with rodents. ILAR J. 43:175–182. 12105384 10.1093/ilar.43.3.175

[phy213904-bib-0053] Nolan, T. E. , M. Loughnane , and A. Jacobson . 2008 Tethered infusion and withdrawal in laboratory animals: a comprehensive look at infusion equipment and materials. ALN Magazine, [Internet]. 2008 Jun 30; Available from: https://www.alnmag.com/article/2008/06/tethered-infusion-and-withdrawal-laboratory-animals

[phy213904-bib-0054] Nyuyki, K. D. , R. Maloumby , S. O. Reber , and I. D. Neumann . 2012 Comparison of corticosterone responses to acute stressors: chronic jugular vein versus trunk blood samples in mice. Stress 15:618–626. 22251167 10.3109/10253890.2012.655348

[phy213904-bib-0055] O'Farrell, L. 1995 Optimal central venous catheter design for long‐term blood sampling in rats. Pennsylvania State University. Pennsylvania State University.

[phy213904-bib-0056] Peternel, L. , Š. Škrajnar , and M. Černe . 2010 A comparative study of four permanent cannulation procedures in rats. J. Pharmacol. Toxicol. Methods 61:20–26. 19622393 10.1016/j.vascn.2009.07.004

[phy213904-bib-0057] Philbin, D. , and C. Coggins . 1980 The effects of anesthesia on antidiuretic hormone. Contemp. Anesthesia Pract. 3:29–38. 7011672

[phy213904-bib-0058] Phillips, W. , W. Stafford , and J. Stuut . 1973 Juglar vein technique for serial blood sampling and intravenous injection in the rat. Proc. Soc. Exp. Biol. Med. Soc. Exp. Biol. Med. 143:733–735. 10.3181/00379727-143-37402 4719460

[phy213904-bib-0059] Picotti, G. , M. Carruba , C. Ravazzani , G. Bondiolotti , and M. Prada . 1982 Plasma catecholamine concentrations in normotensive rats of different strains and in spontaneously hypertensive rats under basal conditions and during cold exposure. Life Sci. 31:2137–2143. 7176812 10.1016/0024-3205(82)90106-0

[phy213904-bib-0060] Popovic, V. , K. M. Kent , and P. Popovic . 1963 Technique of permanent cannulation of the right ventricle in rats and ground squirrels. Proc. Soc. Exp. Biol. Med. Soc. Exp. Biol. Med. 113:599–602. 10.3181/00379727-113-28437 13972284

[phy213904-bib-0061] Popovic, P. , H. Sybers , and V. Popovic . 1968 Permanent cannulation of blood vessels in mice. J. Appl. Physiol. 25:626–627. 5687370 10.1152/jappl.1968.25.5.626

[phy213904-bib-0062] Pritchett‐Corning, K. , Y. Luo , G. Mulder , and W. White . 2011 Principles of rodent surgery for the new surgeon. J. Vis. Exp. (47), e2586. 10.3791/2586 PMC3376945 21248700

[phy213904-bib-0063] Rohde‐Johnson, C. 2016 Tools and techniques for maintaining catheter patency. Lab. Anim. 45:236. 10.1038/laban.1013 27203267

[phy213904-bib-0064] SAI . Press'n Seal can be obtained sterile from SAI [Internet]. Sterilized Glad Press'n Seal. 2018 [cited 2018]. Available from: https://www.sai-infusion.com/products/press-n-seal

[phy213904-bib-0065] Sowemimo‐Coker, S. O. 2002 Red blood cell hemolysis during processing. Transfus. Med. Rev. 16:46–60. 11788929 10.1053/tmrv.2002.29404

[phy213904-bib-0066] Spoelstra, E. , R. Remie , and C. Ince . 2004 Cannulation techniques for multiple blood withdrawal in mice Pp. 11–21 in The physiological genomics of the critically Ill mouse. Springer, US Available from: 10.1007/978-1-4615-0483-2_2

[phy213904-bib-0067] Sutliff, R. L. , E. R. Walp , Y. H. Kim , L. A. Walker , A. M. El‐Ali , J. Ma , et al. 2014 Contractile force is enhanced in aortas from pendrin null mice due to stimulation of angiotensin II‐dependent signaling. PLoS ONE 9:e105101. 25148130 10.1371/journal.pone.0105101 PMC4141771

[phy213904-bib-0068] Suzuki, K. , N. Koizumi , H. Hirose , R. Hokao , N. Takemura , and S. Motoyoshi . 1997 Blood sampling technique for measurement of plasma arginine vasopressin concentration in conscious and unrestrained rats. Lab. Anim. Sci. 47:190–193. 9150500

[phy213904-bib-0069] Tan, R. , A. Dart , and B. Dowling . 2003 Catheters: a review of the selection, utilization and complications of catheters for peripheral venous access. Aust. Vet. J. 81:136–139. 15080425 10.1111/j.1751-0813.2003.tb11074.x

[phy213904-bib-0070] Thrivikraman, K. , R. Huot , and P. Plotsky . 2002 Jugular vein catheterization for repeated blood sampling in the unrestrained conscious rat. Brain Res. Protoc. 10:8494. 10.1016/s1385-299x(02)00185-x 12431707

[phy213904-bib-0071] Tsai, H. , J. Chang , C. Liu , T. Chin , C. Wei , O. Lee , et al. 2014 A newly designed total implantable venous access device in rats for research with high efficiency and low cost. J. Surg. Res. 187:36–42. 24246439 10.1016/j.jss.2013.10.021

[phy213904-bib-0072] Vachon, P. , and J. Moreau . 2001 Serum corticosterone and blood glucose in rats after two jugular vein blood sampling methods: comparison of the stress response. Contemp. Top. Lab. Anim. Sci. 40:22–24. 11560401

[phy213904-bib-0073] Vahl, T. , Y. Ulrich‐Lai , M. Ostrander , C. Dolgas , E. Elfers , R. Seeley , et al. 2005 Comparative analysis of ACTH and corticosterone sampling methods in rats. Am. J. Physiol.‐Endocrinol. Metab. 289:E823–E828. 15956051 10.1152/ajpendo.00122.2005

[phy213904-bib-0074] Van Herck, H. , V. Baumans , C. J. W. M. Brandt , H. A. G. Boere , A. P. M. Hesp , H. A. Van Lith , et al. 2001 Blood sampling from the retro‐orbital plexus, the saphenous vein and the tail vein in rats: comparative effects on selected behavioural and blood variables. Lab. Anim. 35:131–139. 11315161 10.1258/0023677011911499

[phy213904-bib-0075] Verlander, J. W. , K. A. Hassell , I. E. Royaux , D. M. Glapion , M.‐E. Wang , L. A. Everett , et al. 2003 Deoxycorticosterone upregulates PDS (Slc26a4) in mouse kidney: role of pendrin in mineralocorticoid‐induced hypertension. Hypertension 42:356–362. 12925556 10.1161/01.HYP.0000088321.67254.B7

[phy213904-bib-0076] Wall, S. M. , and Y. Lazo‐Fernandez . 2015 The role of pendrin in renal physiology. Annu. Rev. Physiol. 77:363–378. 25668022 10.1146/annurev-physiol-021014-071854

[phy213904-bib-0077] Wickham, R. , S. Purl , and D. Welker . 1992 Long‐term central venous catheters: issues for care. Semin. Oncol. Nurs. 8:133–147. 1621004 10.1016/0749-2081(92)90029-3

[phy213904-bib-0078] Wiersma, J. , and J. Kastelijn . 1985 A chronic technique for high frequency blood sampling/transfusion in the freely behaving rat which does not affect prolactin and corticosterone secretion. J. Endocrinol. 107:285–292. 4067482 10.1677/joe.0.1070285

[phy213904-bib-0079] Williams, W. 1985 A chronically implantable arterial catheter for use in unrestrained small animals. J. Neurosci. Methods 12:195–203. 3982072 10.1016/0165-0270(85)90002-0

[phy213904-bib-0080] Yang, J. , J.‐M. I. Maarek , and D. P. Holschneider . 2016 In vivo quantitative assessment of catheter patency in rats. Lab. Anim. 39:259–268. 10.1258/0023677054307033 PMC4100546 16004684

[phy213904-bib-0081] Yoburn, B. , R. Morales , and C. Inturrisi . 1984 Chronic vascular catheterization in the rat: comparison of three techniques. Physiol. Behav. 33:89–94. 6505057 10.1016/0031-9384(84)90018-0

